# Unilateral Platysma Muscle Rupture as an Effect of Using a Hard Wooden Block for Facial Massage

**DOI:** 10.1055/a-2175-8052

**Published:** 2024-02-07

**Authors:** Kyu Hwa Jung, Eun-Jung Yang, Won Lee

**Affiliations:** 1 Liting Plastic Surgery Clinic, Seoul, South Korea; 2 Department of Plastic and Reconstructive Surgery, Yonsei University College of Medicine, Seoul, South Korea; 3 Yonsei E1 Plastic Surgery Clinic, Anyang, South Korea

**Keywords:** platysma muscle rupture, vigorous facial massage, mass-like lesion

## Abstract

Facial massages are frequently performed to achieve a feeling of freshness, rejuvenation, skin tightening, and delayed onset of wrinkles. However, vigorous massages can induce unexpected symptoms. Here, we present a case of a woman who complained of an asymmetric facial appearance and a mass-like lesion following a long-term facial massage intervention. A facelift incision was performed. Platysma muscle rupture was observed intraoperatively, which was then repaired. To our knowledge, this is the first report of a vigorous facial massage-induced ipsilateral platysma rupture.

## Introduction

Blunt neck traumas only account for approximately 5% of all neck traumas 1 and are most commonly caused by motor vehicle collisions. 2 Only one case of platysma muscle rupture has been reported in the literature. Muscular injuries are often associated with complications or recurrences. 3 These complications depend on the type of injury and are categorized as early, intermediate, or delayed. Muscle rupture usually occurs in sports and develops at the extremities, such as in the rectus femoris muscle, 4 gastrocnemius muscle, 5 or pectoralis major muscle. 6 However, there have been no case reports of facial muscle rupture due to continuous severe stimulation. Herein, we report a case of unilateral platysma muscle rupture caused by facial massage.

## Case

**Fig. 1:**
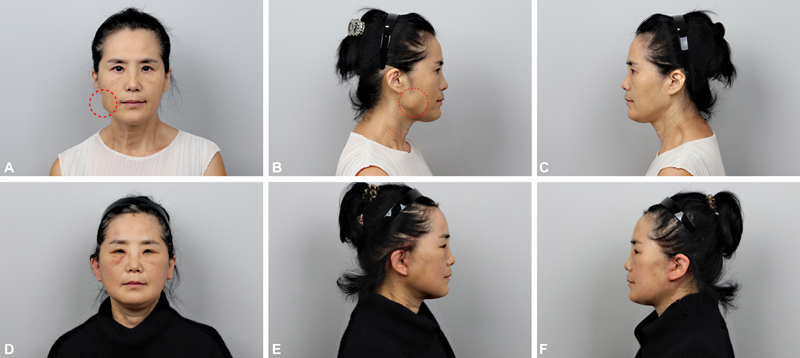
A 63-year-old woman with facial asymmetry and a mass-like lesion on the right lower face, which had grown since she first noticed it 2 months before. ( **A** ) Preoperative frontal view. Mass-like lesion noticed (red dotted circle). ( **B** ) Preoperative right lateral view. Mass-like lesion noticed (red dotted circle). ( **C** ) Preoperative left lateral view. ( **D** ) Postoperative 2 weeks frontal view. ( **E** ) Postoperative 2 weeks right lateral view. ( **F** ) Postoperative 2 weeks left lateral view.

A 63-year-old woman visited our clinic with facial asymmetry and a mass-like lesion on the right lower face, which had grown since she first noticed it 2 months before ( Fig. 1A–C ). The patient received facial massages using a hard wooden block designed for facial massage twice a day for a year. She had no history of surgery or other procedures such as injection of botulinum toxin or fillers or thread lifting. The patient desired removal of the mass for facial symmetry and a bilateral facial lift.

Physical examination revealed a 2 cm × 2 cm movable soft tissue mass, which moved downward during mastication. The mass was located in the subcutaneous layer and could be removed via a conventional face lift operation.

**Fig. 2:**
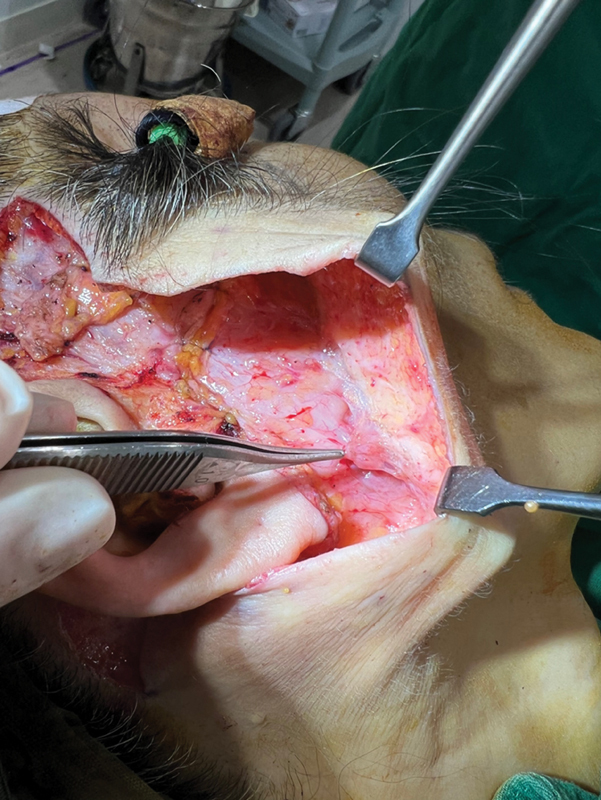
Intraoperatively, the platysma muscle was found to be ruptured near Lore's fascia and had collapsed to form a lump at the border of the mandible.

The patient was prepared for a conventional facelift with tumescent infiltration (2% lidocaine and 1 mL of 1:100,000 epinephrine) into the facial subcutaneous plane. The incision started from the hairline, extended vertically along the anterior helical sulcus and post-tragally, and ended in the postauricular area. An incision was made in the subcutaneous layer, and the superficial musculoaponeurotic system (SMAS) was exposed. Intraoperatively, the platysma muscle was found to be ruptured near Lore's fascia and had collapsed to form a lump at the border of the mandible ( Fig. 2 ). The platysma was elevated as a muscle flap, and a fully released masseteric retaining ligament was used for the repair. The end of the muscle was sutured near Lore's fascia using size 3–0 vicryl sutures to maintain muscle tension. An extended SMAS dissection was also performed and elevated in the superolateral direction. Skin flap redraping was performed, and the remnant skin was excised. Skin closure was performed using size 6–0 nylon sutures. No mass-like structure was detected postoperatively. Postoperative 2 weeks' photographs were taken ( Fig. 1D–F ). The patient provided written informed consent.

## Discussion

We presented a case of platysma muscle rupture caused by facial massage. The platysma muscle attaches to the lower border of the mandible and mandibular septum and merges with the facial muscles around the lower lip. It originates from the upper portion of the thorax anterior to the clavicle at the subcutaneous tissue of the subclavicular region and the pectoralis. The lymphatics of the anterior neck lie above the platysma muscle, and manual lymphatic drainage can be performed to reduce edema and pain as well as enhance the range of motion and patients' quality of life. 7 Many people undergo facial massages to feel fresh and rejuvenated, tighten the skin, and delay the onset of wrinkles. 8 Meridian facial massages are commonly performed in Korea for skin rejuvenation and facial lifting and to decrease the face size. 9 Several people undergo these procedures although their effectiveness has not been scientifically proven. In our case, the patient had used a hard wooden block for facial massage for a year. The use of *gua sha* and jade rollers originates from ancient China. 10 Their use can stimulate a powerful facial massage but cause complications. To the best of our knowledge, this is the first reported case of platysma muscle rupture caused by frequent facial massages.

Vigorous facial massage can induce platysma muscle rupture, although this is rare. We report a case of unilateral platysma muscle rupture caused by facial massage. It is necessary to further investigate this unique etiology and implement appropriate muscle sutures based on the intraoperative findings.

## References

[1] Levy D Neck trauma. Emedicine.1–11Accessed June 19, 2006 at:www.emedicine.com/emerg/topic331.htm

[2] Britt L D Peyser M B Chapter 22. Trauma. 5th ed; 2004 445 458

[3] Alessandrino F Balconi G Complications of muscle injuries J Ultrasound 2013 16 04 215 222 24432177 10.1007/s40477-013-0010-4 PMC3846951

[4] Temple H T Kuklo T R Sweet D E Gibbons C L Murphey M D Rectus femoris muscle tear appearing as a pseudotumor Am J Sports Med 1998 26 04 544 548 9689376 10.1177/03635465980260041301

[5] McClure J G Gastrocnemius musculotendinous rupture: a condition confused with thrombophlebitis South Med J 1984 77 09 1143 1145 6484684 10.1097/00007611-198409000-00023

[6] Wolfe S W Wickiewicz T L Cavanaugh J T Ruptures of the pectoralis major muscle. An anatomic and clinical analysis Am J Sports Med 1992 20 05 587 593 1443329 10.1177/036354659202000517

[7] Provencher A M Giguère-Lemieux É Croteau É Ruchat S M Corbin-Berrigan L A The use of manual lymphatic drainage on clinical presentation of musculoskeletal injuries: a systematic review Complement Ther Clin Pract 2021 45 101469 34343761 10.1016/j.ctcp.2021.101469

[8] Khanna N Datta Gupta S Rejuvenating facial massage—a bane or boon? Int J Dermatol 2002 41 07 407 410 12121555 10.1046/j.1365-4362.2002.01511.x

[9] Lee Y O Jung I K A study on the change of facial size by meridian scrapping massageKor J Aesthet Cosmetol 2010;8(04):

[10] Hamp A Anderson J Laughter M R Gua-sha, jade roller, and facial massage: are there benefits within dermatology? J Cosmet Dermatol 2023 22 02 700 703 36170573 10.1111/jocd.15421

